# Influence of CReatine Supplementation on mUScle Mass and Strength After Stroke (ICaRUS Stroke Trial): A Randomized Controlled Trial

**DOI:** 10.3390/nu16234148

**Published:** 2024-11-29

**Authors:** Juli T. Souza, Marcos F. Minicucci, Natália C. Ferreira, Bertha F. Polegato, Marina P. Okoshi, Gabriel P. Modolo, Filipe W. Leal-Pereira, Bethan E. Phillips, Philip J. Atherton, Kenneth Smith, Daniel J. Wilkinson, Adam L. Gordon, Suzana E. Tanni, Vladimir E. Costa, Maria F. Fernandes, Silméia G. Bazan, Leonardo M. Zornoff, Sérgio R. Paiva, Rodrigo Bazan, Paula S. Azevedo

**Affiliations:** 1 Department of Internal Medicine, Medical School, São Paulo State University (UNESP), Botucatu 18618-970, SP, Brazil; marcos.minicucci@unesp.br (M.F.M.); nataliafactori@hotmail.com (N.C.F.); bertha.polegato@unesp.br (B.F.P.); marina.okoshi@unesp.br (M.P.O.); filipe.leal@unesp.br (F.W.L.-P.); suzana.tanni@unesp.br (S.E.T.); mariafernandaprimo@hotmail.com (M.F.F.); sgz.bazan@unesp.br (S.G.B.); leonardo.zornoff@unesp.br (L.M.Z.); sergio.paiva@unesp.br (S.R.P.); schmidt.azevedo@unesp.br (P.S.A.); 2 Department of Neuroscience and Mental Health, Medical School, São Paulo State University (UNESP), Botucatu 18618-970, SP, Brazil; gpmodolo@gmail.com (G.P.M.); rodrigo.bazan@unesp.br (R.B.); 3 MRC-Versus Arthritis Centre for Musculoskeletal Ageing Research (CMAR) & NIHR Nottingham Biomedical Research Centre, University of Nottingham Medical School, Derby Uttoxeter Road, Derby DE22 3DT, UK; beth.phillips@nottingham.ac.uk (B.E.P.); philip.atherton@nottingham.ac.uk (P.J.A.); ken.smith@nottingham.ac.uk (K.S.); d.wilkinson@nottingham.ac.uk (D.J.W.); 4 Wolfson Institute of Public Health, Queen Mary University of London, London E1 4NS, UK; adam.gordon@qmul.ac.uk; 5 Academic Centre for Healthy Ageing, Whipps Cross Hospital, Barts Health NHS Trust, London E1 1BB, UK; 6 Stable Isotopes Center, São Paulo State University (UNESP), Institute of Biosciences, Botucatu 18618-970, SP, Brazil; vladimir.costa@unesp.br

**Keywords:** stroke, creatine, muscle mass, older people, progranulin, sarcopenia

## Abstract

Background/Objectives: The acute phase of stroke is marked by inflammation and mobility changes that can compromise nutritional status. This study was a randomized, double-blind, placebo-controlled trial evaluating the effectiveness of creatine supplementation for older people during seven days of hospitalization for stroke compared to usual care. Method: The primary outcome measures were changes in functional capacity, strength, muscle mass, and muscle degradation. The secondary outcomes were changes in serum biomarkers related to inflammation, fibrosis, anabolism, and muscle synthesis. In addition, a follow-up 90 days after the stroke verified functional capacity, strength, quality of life, and mortality. Following admission for an acute stroke, participants received either creatine (10 g) or a visually identical placebo (10 g) orally twice daily. Both groups received supplementation with protein to achieve the goal of 1.5 g of protein/kg of body weight/day and underwent daily mobility training during seven days of hospitalization. Results: Thirty older people were included in two similar groups concerning baseline attributes (15—treatment/15—placebo). Conclusions: Creatine supplementation did not influence functional capacity, strength, or muscle mass during the first 7 days or outcomes 90 days after stroke. There were no serious adverse events associated with creatine supplementation. However, it decreased progranulin levels, raising a new possibility of creatine action. This finding needs further exploration to understand the biological significance of creatine–progranulin interaction.

## 1. Introduction

Stroke is one of the principal causes of death and disability across the world [1,2]. Long-term disability is the most frequent complication after stroke, with 50% of patients experiencing hemiparesis and 30% unable to walk without assistance [3,4,5,6,7]. Coupled with this, patients become more susceptible to involuntary weight, muscle, and strength loss following stroke. For example, three weeks after stroke-affected individuals experience a significant reduction in muscle mass, which is usually accompanied by fat deposition in the limbs, resulting in decreased strength and muscle function, is sometimes referred to as “stroke-related sarcopenia” [8]. In fact, muscle damage starts as soon as four hours after a stroke [9], with paralysis and inflammation as the leading causes [10].

After an acute injury, such as a stroke, the body reacts with the metabolic response to stress, marked by inflammation and immune and hormonal responses. This yields hypermetabolism and catabolism, contributing to weight, muscle, and strength loss. It is challenging to stop the nutritional decline, especially within the first week after an acute injury [11]. Therefore, it is worthwhile to investigate nutraceuticals that could act as anti-inflammatory and anabolic agents.

Creatine has been studied concerning its potential to promote muscle hypertrophy and improve muscle strength and performance [12]. Creatine has also been suggested to have the potential to mitigate deterioration in many physiological parameters that contribute to sarcopenia and cachexia, including alterations in cellular bioenergetics, anabolism, and inflammation [13,14]. These effects are realized through the importance of Creatine–Phosphocreatine (PCr) to tissues with high and unsteady energy requirements, such as muscle, heart, and brain, because it provides a ready-release high-energy phosphate pool for maintaining adequate ATP levels [15]. As an anti-inflammatory agent, creatine reduced NFκB, TNF-α, IL-6, and tall-like receptors in some studies, but not all [16]. The heterogeneity of these studies’ population, the difference in the grade of inflammation, and the association with physical exercises could explain, in part, the divergent results that need to be further elucidated [16].

Novel inflammatory and hormonal markers have been studied in muscle disorders such as frailty and sarcopenia but not specifically in muscle dysfunction induced by stroke. Across various populations, the following biomarkers are related to inflammation in addition to other aspects related to skeletal muscle function: (i) Growth/Differentiation Factor-15 (GDF-15) is a biomarker of mitochondrial function and muscle apoptosis; (ii) Progranulin (PGRN) is related to tissue regeneration; (iii) Metalloprotease-2 (MMP-2) and 9 (MMP-9) are related to extracellular matrix degradation; and (iv) type III procollagen peptide (P3NP) is associated with fibrosis and changes in muscle mass [17,18]. In addition, Interleukin-6 (IL-6) and insulin-like growth factor 1 (IGF-1) are each markers of anabolism, while 3-methylhistidine (3-MH) is related to muscle degradation [19,20].

Considering the clear detrimental impact of skeletal muscle dysfunction after stroke, nutritional strategies must attenuate or mitigate these declines. To our knowledge, no studies have evaluated the impact of creatine supplementation in the acute phase of stroke or its role in attenuating muscle dysfunction or inflammatory and hormonal biomarkers after stroke [14].

Against this background, this study aimed to investigate the effectiveness of creatine supplementation on the functional capacity, strength, and change in muscle mass of older people during hospitalization for stroke, compared to usual care. It also assessed the impact of creatine supplementation on inflammation biomarkers, muscle synthesis, and muscle degradation. Finally, a follow-up 90 days after the stroke evaluated the impact of stroke and creatine supplementation on longer-term functional capacity, strength, quality of life, and mortality.

## 2. Methods

### 2.1. Trial Design

This was a single-center, randomized, double-blind, parallel-group trial that included older people in the acute phase (within 24 h) of ischemic stroke to determine the impact of creatine supplementation compared to maltodextrin (placebo) on skeletal muscle parameters. The trial duration for the individual participants was approximately 90 days, and each subject attended a maximum of three visits: (1) 24 h after stroke (Moment 1); (2) 7th day of hospitalization (Moment 2); and (3) 90 days after stroke (Moment 3). A protocol paper outlines the full details of this trial [21].

### 2.2. Participants and Eligibility Criteria

Men and women aged 60 years and older who were diagnosed with ischemic stroke, without previous disability (Modified Rankin Scale [mRs] ≤ 2), and who were able to provide informed consent were recruited in the first 24 h after the stroke. Exclusion criteria included patients with hemodynamic instability, those requiring mechanical ventilation, and those with pacemakers and/or metal prostheses due to interference in bioimpedance results. Previous kidney disease (dialysis, end-stage renal disease, or creatinine clearance ≤ 30 mL/min/1.73 m^2^ assessed within 24 h before recruitment), a history of malabsorptive gastrointestinal surgery, parenteral nutrition, and/or allergies or intolerance to any component of the study products were also exclusion criteria. Participants who were found to be intolerant of supplements could not stay in the Comprehensive Stroke Center (CSC), or who withdrew their consent were excluded from this study. During the study, adding a nurse to the research team made it possible to include individuals who used enteral tube feeding (amendment opinion number 5.456.441 of 8 June 2022).

This study was conducted at the CSC of the Clinical Hospital of Botucatu Medical School, Brazil, with 90-day follow-up visits at the Neurovascular Disease Outpatient Clinic of the same institution. Recruitment was carried out by the daily monitoring of admissions to the CSC, with potential participants screened against inclusion/exclusion criteria and invited to participate in this study. This study was approved by the Research Ethics Committee of Sao Paulo State University according to the Consubstantiated Opinion no. 2.421.189 (CAAE: 79844317.3.0000.5411—7 December 2017), and all participants signed an Informed Consent Form.

### 2.3. Interventions

Creatine supplementation was administered in the treatment group (one sachet containing 10 g of creatine—twice daily), and the placebo group received maltodextrin, a complex carbohydrate (one sachet containing 10 g of maltodextrin—twice daily). All sachets were the same color and size. All participants received additional supplementation with powdered milk protein serum isolate to achieve a daily protein intake of 1.5 g/kg of body weight. Additional supplementation was given during the 7-day hospitalization period, starting in the first 24 h after stroke.

Physical rehabilitation started for all participants after the first 24 h after the stroke, and the intensity was proportional to each individual’s tolerance. Early mobility training was performed according to the current rehabilitation guidelines for stroke patients and applied by the team of physiotherapists specialized in stroke at the Rehabilitation and Physiotherapy Unit of the Clinical Hospital of Botucatu Medical School, Brazil [22,23].

All participants were followed up often and stimulated to intake all meals and supplements. Similar foods were substituted when necessary into participants’ diets to increase food and supplementation acceptance during the intervention period. The research team kept empty sachets for checking daily.

At hospital discharge, all participants were instructed on healthy eating habits, such as fruits, vegetables, whole grains, adequate water intake, and avoiding processed foods. Health food preparation was encouraged (e.g., avoiding frying by immersion in oil). All instructions were based on the Ministry of Health’s Dietary Guidelines for the Brazilian Population [24].

### 2.4. Outcomes

The primary outcomes were the change in functional capacity assessed by mRs [25]; muscle strength assessed by handgrip [26]; change in muscle mass evaluated by ultrasonography (muscle thickness of the rectus femoris and biceps brachii muscles in both stroke-affected and unaffected limbs) using a BodyMetrix BX-2000 ultrasound device with a 2.5 MHz transducer (Intelametrix, Livermore, CA, USA) [27] and multifrequency electrical bioimpedance (fat-free mass index and appendicular muscle mass index) (Seca model mBCA 525, Hamburgo, Germany) [28], and muscle degradation by the serum biomarker 3-MH [evaluated in 2 ways: labeled isotope D3-methylhistidine using liquid chromatography-mass spectrometry and ELISA (Kit EH4250—3MH—Wuhan Fine Biotech, Wuhan, China)] [20].

The secondary outcomes were changes in serum biomarkers related to inflammation, fibrosis, anabolism, and muscle synthesis (IL-6 (Kit E-OSEL-H001-IL 6—Elabscience, Houston, TX, USA), GDF-15 (Kit E-EL-H0080 GDF15—Elabscience-USA), PGRN (Kit E-EL-H1578-PGRN—Elabscience, Houston, TX, USA), MMP-2 (Kit E-EL-H1445-MMP2—Elabscience, Houston, TX, USA), and MMP-9 (Kit E-EL-H6075-MMP9—Elabscience, Houston, TX, USA), in IGF-1 (Kit E-EL-H0086 IGF-1—Elabscience, Houston, TX, USA), and P3NP (Kit E-EL-H0182- PC III—Elabscience, Houston, TX, USA).

All the evaluations described above were conducted within 24 h after the stroke and repeated on the seventh day of hospitalization.

### 2.5. Follow-Up

A follow-up 90 days after the stroke was performed to establish functional capacity assessed by mRs and the Timed Up and Go test [25,29]; muscle strength assessed by handgrip and the 30 s chair stand test score [30]; quality of life was assessed by the EuroQol-5D questionnaire (registration number: 51687 In: https://customer.euroqol.org, accessed on 23 June 2023) [31], and mortality data were collected from medical records or phone calls. Handgrip strength and mRs were assessed within the first 24 h after the stroke, on the 7th day of hospitalization, and 90 days after the event. However, the 30 s chair stand test and the Timed Up and Go test were performed on the 7th day and after 90 days because patients are not allowed to stand up during the first 24 h after stroke.

### 2.6. Sample Size

A previous study analyzed the effects of creatine supplementation in 18 healthy older people, in which ten patients were allocated to the treatment group, and eight to the placebo group, and an improvement in muscle strength, weight, and fat-free mass in the group that received supplementation was observed [32]. In addition, a meta-analysis with an average of 10 patients in each arm per study showed that creatine supplementation is effective in lower limb strength performance for exercises lasting less than 3 min [33]. Considering this, we included 50% more participants in our sample, totaling 15 patients in each group. At the end of this pilot study investigating the primary outcome in clinical parameters, a post hoc power calculation was performed, and it was found that, for these outcomes, the post hoc power of the sample size was low, directly impacting the evaluation of the primary hypothesis. However, a secondary outcome was identified that could guide pathways related to supplementation mechanisms that need to be studied further.

### 2.7. Randomization

Screening and enrolment were performed at the same time for all subjects. Subjects were randomized 1:1 to treatment or placebo groups using a cell phone application (Randomizer), which created a random placebo and treatment sequence for blocks of 10 participants. No stratification was used. One person was responsible for randomizing and separating sachets containing study material into sealed envelopes identified with the number of each participant. This person did not have access to research participants at any time.

### 2.8. Blinding

Participants and research team members responsible for administering supplements, performing biochemical analyses, and conducting protocol assessments were blinded to the intervention. Creatine and placebo had the same appearance, and sachets were identical. The administration method for both products was the same to avoid breaking the blinding. Breaking the blinding would have been allowed if any participant had a serious adverse effect related to the supplementation, such as an allergic reaction, but was not needed during this study.

### 2.9. Statistical Analysis

All study participants consumed more than 85% of the sachets offered. The baseline was defined at the point of randomization and the end of the trial at follow-up 90 days after the stroke. Baseline categorical data were summarized by the treatment group using the number and percentages of subjects. The denominator for calculating percentages was the number of subjects in the analysis set.

The continuous characteristic variables between groups were compared using the Student’s *t*-test or the Mann–Whitney test for parametric and non-parametric data, respectively, and categorical variables using Chi-square or Fisher’s exact test. The Sigma Plot 12.0 statistical program (Dundas Software LLC, Germany) was used.

Mixed regression models assessed differences in serum biomarkers, muscle mass, and strength between groups and over time. Data were modeled according to the GMM (Generalized Mixed Models) using normal and gamma distributions. The chosen model had the lowest AIC (Akaike Information Criterion) value. Post hoc analysis (Bonferroni) was performed for all statistically significant variables. The Jamovi statistic program (Version 2.3) (Computer Software, Australia) was used for these analyses.

All statistical tests were two-sided and performed on a 5% significance level.

The Kaplan–Meier survival curve was used to present survival in the groups within 90 days. Considering day zero as the day of randomization and entry into this study, patients reported the final date that they had each outcome. P-values were obtained through proportional Cox regression models, and a value of 5% was considered statistically significant.

## 3. Results

### 3.1. Recruitment and Characteristics of the Participants

**Figure 1:**
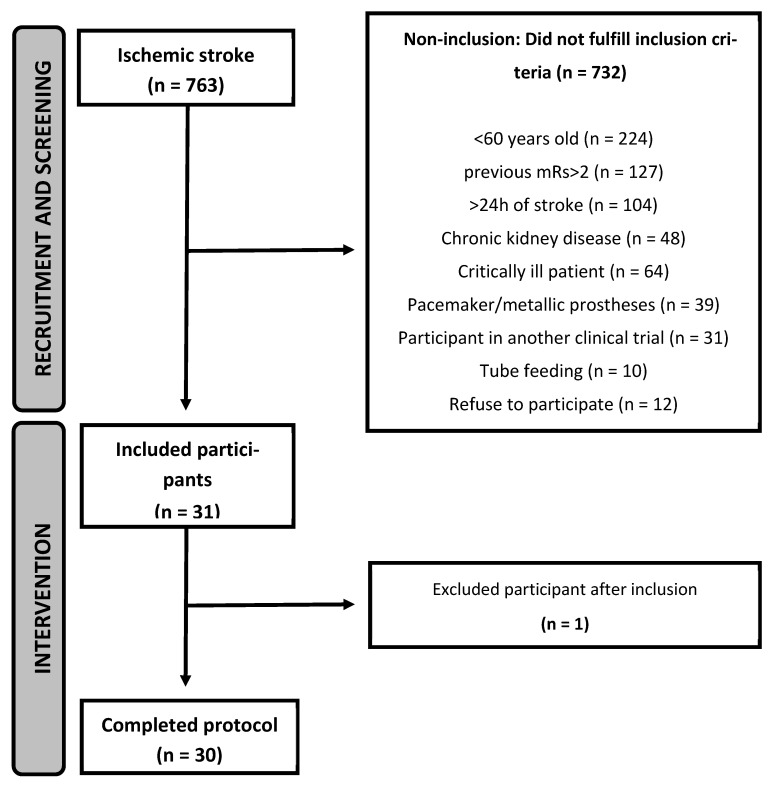
Flowchart of inclusion of participants in the ICaRUS stroke trial from March 2019 to May 2023.

Recruitment started in March 2019 and ended in May 2023. During this period, 763 patients had a diagnosis of ischemic stroke at the study site, and 31 were included in this study. One participant was excluded shortly after inclusion as he was diagnosed with COVID-19 and needed to be transferred to another unit. Thus, 30 participants followed the study protocol, with 15 participants in each group (Figure 1). The last follow-up visit occurred in August 2023. The trial ended when N was reached. It was not possible to screen 66 patients who had an ischemic stroke at the study site during the study period because of infection control measures in place in the hospital during outbreaks of COVID-19.

The general and neurological characteristics of the participants at the time of inclusion in this study were similar in both groups (Table 1).

### 3.2. Primary Outcomes

During the seven days of hospitalization, creatine supplementation did not influence functional capacity assessed by mRs, muscle strength assessed by handgrip, changes in muscle mass assessed by electrical bioimpedance and muscle ultrasound, or muscle degradation assessed by 3MH analysis, when compared to the placebo group. Findings for these primary outcomes are summarized in Table 2.

### 3.3. Secondary Outcomes

**Figure 2:**
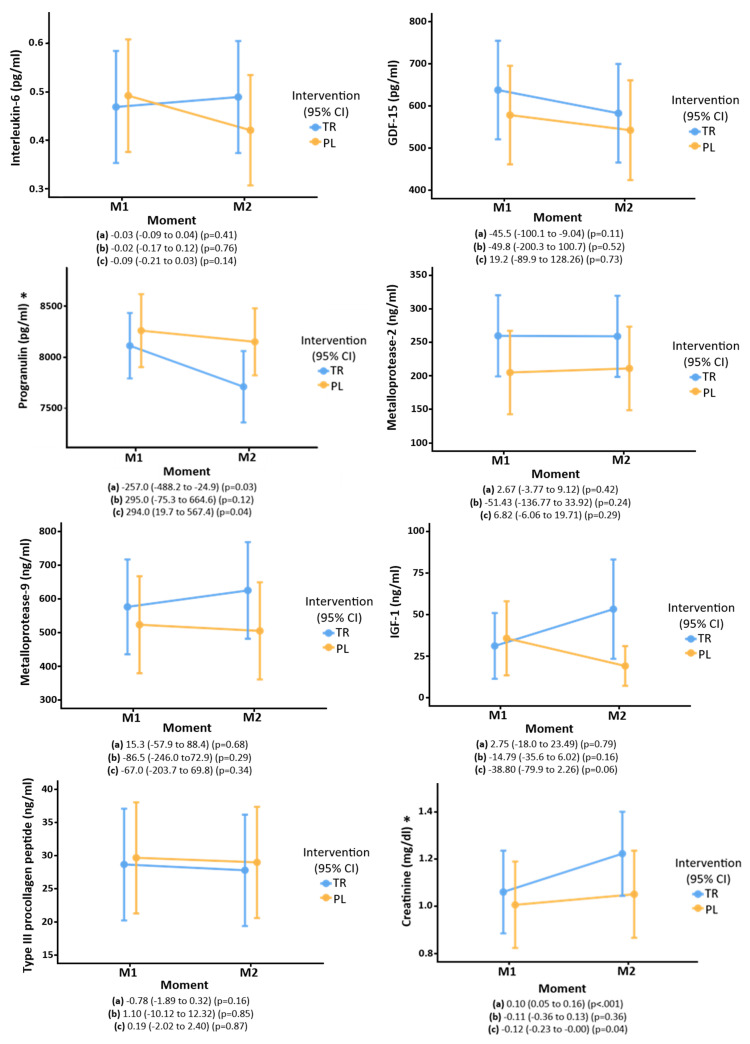
Biochemical markers of older people who participated in the ICaRUS stroke trial in the first 24 h after stroke and on the 7th day of intervention with creatine versus placebo: (**a**) Moment: estimate (95%CI) (P). (**b**) Treatment: estimate (95%CI) (P). (**c**) Interaction of time × treatment: estimate (95%CI) (P). TR: Treatment. PL: Placebo. M1: Moment 1 (24 h after stroke).M2: Moment 2 (7th day of hospitalization). GDF-15: Growth/Differentiation Factor-15. IGF-1: Insulin-like growth factor 1. Performed generalized mixed models using normal and gamma distributions. * Post hoc analysis (Bonferroni) showed a difference between the moments only in the intervention group for progranulin [(difference = 403.3) (*p* = 0.02) and creatinine [(difference = −0.16) (*p* = 0.001)].

Regarding the biochemical tests, creatine supplementation was associated with higher serum creatinine levels (*p* = 0.04) and decreased PGRN (*p* = 0.04) compared to the placebo group. No effect of treatment was seen on other biomarkers (Figure 2).

### 3.4. Follow-Up Visit

**Figure 3:**
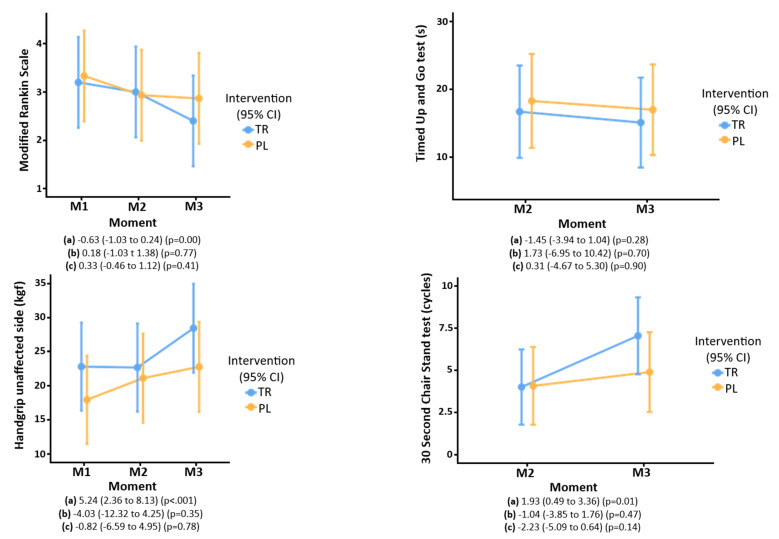
Functional capacity and muscle strength evaluated by the modified Rankin scale, Timed Up and Go test, handgrip, and 30 s chair stand test of older people who participated in the ICaRUS stroke trial. (**a**) Moment: estimate (95%CI) (P). (**b**) Treatment: estimate (95%CI) (P). (**c**) Interaction of time × treatment: estimate (95%CI) (P). TR: Treatment. PL: Placebo. M1: Moment 1 (24 h after stroke). M2: Moment 2 (7th day of hospitalization). M3: Moment 3 (90 days after stroke). Performed generalized mixed models using normal and gamma distributions.

Ninety days after the stroke, functional capacity (assessed by the mRs and Timed Up and Go test), and muscle strength (assessed by handgrip and the 30 s chair stand test) improved, independent of creatine supplementation (Figure 3).

**Figure 4:**
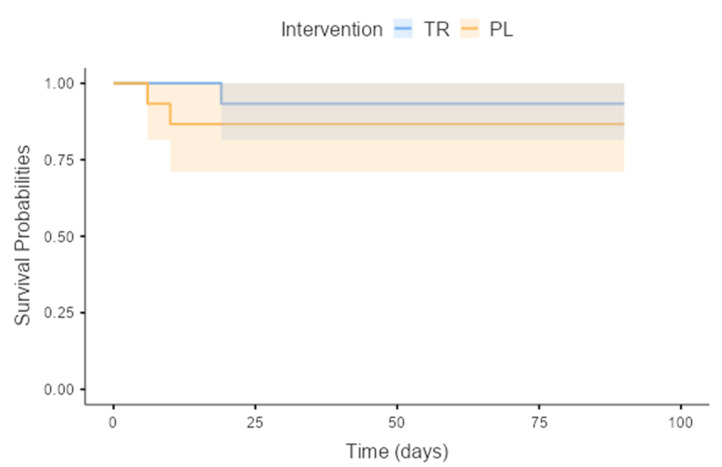
Kaplan–Meier survival curve of the older people during participation in the Icarus stroke trial. TR: Treatment; PL: Placebo. (*p* = 0.52).

The treatment [8.0 (7.0–10.0)] and placebo [7.0 (6.0–10.5)] groups (*p* = 0.54) were similar regarding quality of life 90 days after stroke (treatment group 73.8 ± 22.2 and placebo group 65.6 ± 27.5) (*p* = 0.41) as assessed by EuroQol score. Three participants died during the study period. According to Cox regression, there was no difference in mortality between treatment and placebo groups (*p* = 0.52) over the 90-day follow-up period, regardless of age, sex, or stroke severity (Figure 4).

### 3.5. Safety

**Figure 5:**
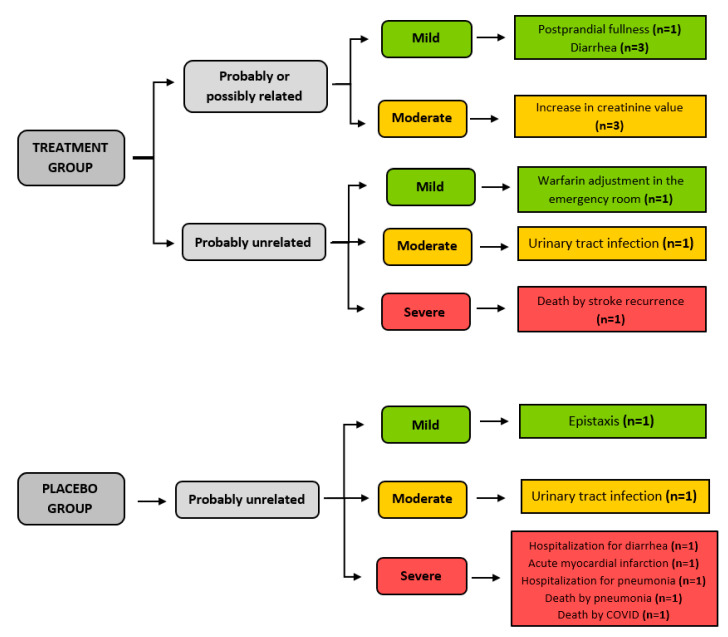
Adverse events that occurred during the participation of older people in the ICaRUS stroke trial.

Concerning adverse events during this study, three participants who received creatine had diarrhea, and one complained of postprandial fullness after consuming the creatine supplement, with rapid improvement after adjusting the administration time to be further removed from the subsequent meal. Three participants in the treatment group had significantly increased serum creatinine levels (above 60% of the initial value) at the end of the seven days of intervention, with rapid resolution after hydration (Figure 5).

## 4. Discussion

This small but novel study demonstrates that supplementing creatine in the acute phase of stroke is feasible and safe. After a short (7-day) course of treatment, creatine supplementation did not impact functional capacity, strength, muscle mass, or muscle degradation. It also did not change inflammatory and hormonal dosages such as IGF-1, IL-6, GDF-15, Pro-collagen type 3, and metalloproteases. However, it did reduce PGRN. This finding raises a new pathway of creatine action in patients after stroke that further studies need to explore.

In this study, supplementation with 20 g/day of creatine, while also ensuring adequate protein intake for seven days after acute stroke, appeared safe considering the observed adverse events, most of which (including all categorized as severe) were deemed not treatment-related. An increase in serum creatinine values was observed in the group treated with creatine, indicating a biological effect even in our short intervention period. Around 2% of endogenous creatine is excreted daily as creatinine, so the increase in serum creatinine reflects an increase in ingested creatine [14].

As creatine is a compound with anabolic and anti-inflammatory properties, we tested it in a challenging situation: the acute phase of stroke. A stroke can be considered an acute injury in which a metabolic response is observed in both the brain and systemically [34,35]. Therefore, this study’s primary and secondary outcomes were related to muscle phenotyping, function, degradation, performance and metabolism, and systemic inflammation [19,36].

In this study, creatine did not change functional capacity, muscle strength, mass, or degradation. This lack of effect on these parameters may have resulted partly from the difficulty of adequately exercising [37]. Although our participants did engage with the early mobility training protocol, this was not high intensity, as seen in other studies assessing the impact of creatine supplementation in healthy populations who reported functional benefits [38,39]. In addition, stroke recruits fast-twitch fibers whose energy comes predominantly from anaerobic metabolism and are more susceptible to fatigue. Therefore, creatine might not have been enough to counteract the burden of the acute stress of stroke on this type of fiber [8,9].

A marked difference between our study and those conducted in ‘healthy’ individuals is the inflammatory scenario in post-stroke patients [40,41]. With inflammation known to promote catabolism and mitigate anabolism [34,35], it is plausible that our lack of phenotypic changes (i.e., muscle mass and physical function) was due to inflammation-inducing an anabolic blunt, reducing the effect of creatine supplementation. We did not find clinical studies where creatine was supplemented in acute stress situations, such as stroke. In cachexia patients, which encompasses low-grade chronic inflammation, controversial findings were observed, with some studies with positive effects and others with no impact. Following our findings, the authors suggested that the inconsistent results are explained, for example, by small sample sizes, no proper exercise intervention, and the length and dosage of creatine used [14].

In the present study, creatine reduced PGRN in the acute phase of stroke. Previous research has shown that the effects of PGRN demonstrate a “U”-shaped curve. For example, although PGRN has been shown to exert anti-inflammatory effects, and its deficiency is associated with neurological diseases [36,42], excess PGRN is associated with poor prognosis in cancer and stroke [43]. This disparity is partly explained by neutrophils secreting an elastase during an acute inflammatory reaction, which cleaves PGRN into granulin, a pro-inflammatory peptide, potentially exacerbating the inflammation [42]. For example, in an experimental study where cerebral embolization induced stroke in animal models, the levels of PGRN and granulin were increased in the cerebral cortex. The ischemic injury was suppressed after the treatment with a neutrophil elastase inhibitor that reduced the conversion of PGRN into granulin [44]. In addition, studies with humans in the acute phase of stroke showed that PGRN was increased. Those with a more severe stroke and significant functional disability had even higher PGRN levels. Further, there was an association between increased PGRN, mortality, and worse functional outcomes in this population [45,46].

In this study, a reduction in PGRN was observed in both groups, but it was statistically more pronounced in the treatment group. The average PGRN after seven days of creatine administration reduced to the normal range, similar to the pattern previously seen by Xie et al. [46]. It is challenging to interpret this relationship between creatine and PGRN in the context of stroke. Since there is a suggestion that PGRN could be an inflammatory biomarker of poor prognosis in acute stroke [46], the modulation of its level could raise the hypothesis of an anti-inflammatory role of creatine in the acute phase of stroke. Further, an anti-progranulin antibody (anti-PGRN monoclonal antibody A23) has been shown to reduce tumor growth and stabilize disease in cases of hepatocellular carcinoma in non-candidates for surgery [43].

Thus, one of the novelties of the present study is showing the potential of creatine to modulate PGRN in the acute phase of stroke. This paves the way for new studies to identify the role of PGRN in the prognosis of stroke and whether modulating its serum levels could benefit it.

MMP-2, MMP-9, IGF-1, PN3P, IL-6, and GDF-15 were also studied, but creatine did not influence this parameter. Regarding the 90-day follow-up, according to Cox’s regression, there was no difference in mortality between treatment and placebo groups, regardless of age, sex, or stroke severity. There was evidence of recovery of functional capacity and strength on the unaffected side, but this was independent of creatine supplementation. There was also no difference between the treatment and placebo groups regarding quality of life. However, it should be acknowledged that findings related to quality of life are generally associated with the individual’s degree of disability [47].

The limitations of this study include a small sample size, a short intervention period, and difficulty implementing exercise in the immediate post-stroke period. However, this study also has several strengths. It is a pragmatic clinical trial of an exploratory type conducted with methodological rigor and the careful analysis of multiple variables within the acute phase after stroke. In addition, the deep phenotyping of muscle parameters and several serum biomarkers was performed in two moments (admission and seven days), with a 90-day follow-up of longer-term impacts.

## 5. Conclusions

In conclusion, creatine supplementation at a dose of 20 g/day for 7 days proved safe, with good tolerance and acceptability. It did not influence functional capacity, strength, or muscle mass during the first 7 days or outcomes 90 days after stroke. However, it decreased progranulin levels, raising a new possibility of creatine action. This finding needs further exploration to understand the biological significance of creatine–progranulin interactions.

## Figures and Tables

**Table 1:** Baseline general and neurological characteristics of older people hospitalized for ischemic stroke who participated in the ICaRUS stroke trial and were randomized to the treatment or placebo groups.

	Treatment (n = 15)	Placebo (n = 15)	*p*
Male sex, N (%)	10.0 (66.7)	10.0 (66.7)	0.70
Age (years)	69.0 (64.0–79.0)	66.0 (63.0–73.0)	0.88
NRS 2002 ≥ 3, N (%)	8.0 (53.3)	9.0 (60.0)	1.00
Diabetes mellitus, N (%)	4.0 (26.7)	6.0 (40.0)	0.70
Arterial hypertension, N (%)	10.0 (66.7)	12.0 (80.0)	0.68
Dyslipidemia, N (%)	5.0 (33.3)	6.0 (40.0)	1.00
Previous MI, N (%)	4.0 (26.7)	2.0 (13.3)	0.65
Previous stroke, N (%)	1.0 (6.7)	2.0 (13.3)	1.00
Thrombolysis, N (%)	6.0 (40.0)	6.0 (40.0)	0.71
Insulin use, N (%)	5.0 (33.3)	3.0 (20.0)	0.68
Statin use, N (%)	5.0 (33.3)	2.0 (13.3)	0.39
Dysphagia, N (%)	3.0 (20.0)	6.0 (40.0)	0.43
FOIS, N (%)	6.0 (40.0)	6.0 (40.0)	0.15
ASPECTS score	10.0 (8.0–10.0)	8.0 (8.0–9.0)	0.13
NIHSS at admission	7.0 (4.0–13.0)	8.0 (6.0–12.0)	0.55
Bamford classification			0.85
LACS, N (%)	7.0 (23.3)	6.0 (20.0)	
PACS, N (%)	4.0 (13.3)	6.0 (20.0)	
TACS, N (%)	2.0 (6.7)	1.0 (3.3)	
POCS, N (%)	2.0 (6.7)	2.0 (6.7)	
Toast classification			0.41
Cardioembolism, N (%)	3.0 (10.0)	4.0 (13.3)	
Large-vessel, N (%) atherothrombosis, N (%)	4.0 (13.3)	2.0 (6.7)	
Undetermined causes, N (%)	8.0 (26.7)	7.0 (23.3)	
Small-vessel disease, N (%)	0.0 (0.0)	2.0 (6.7)	

NRS: nutritional risk screening (to verify the presence of nutritional risk at the baseline visit); MI: acute myocardial infarction; FOIS: functional oral intake scale (to verify differences in diet consistency at baseline visit); ASPECTS: Alberta Stroke Program Early CT Score; NIHSS: National Institutes of Health Stroke Scale; LACS: lacunar syndrome; PACS: partial anterior circulation syndrome; TACS: total anterior circulation syndrome; POCS: posterior circulation syndrome. Performed Chi-square or Fisher Exact test to evaluate categorical variables; the results were expressed as numbers and percentages. Performed Mann–Whitney for age, ASPECTS score, and NIHSS; the results were expressed as the median and 25th–75th percentiles.

**Table 2:** Influence of creatine supplementation, for seven days, on functional capacity, muscle strength, mass, and degradation in older people hospitalized for stroke (n = 30).

Outcomes			95% Confidence Interval		
	Variables	Estimate	Lower	Upper	*p*
Modified Rankin Scale	Moment	−0.30	−0.55	−0.05	0.03
	Treatment	0.03	−1.26	1.33	0.96
	Interaction of time × treatment	−0.20	0.71	0.31	0.44
Handgrip unaffected side (kgf)	Moment	1.45	−0.40	3.29	0.14
	Treatment	−3.29	−12.41	5.84	0.49
	Interaction of time × treatment	3.16	−0.53	6.85	0.10
Handgrip affected side (kgf)	Moment	0.59	−1.90	3.08	0.64
	Treatment	−0.34	−10.36	9.67	0.95
	Interaction of time × treatment	−0.02	−5.00	4.97	0.99
FFMI (kg/m^2^)	Moment	−0.71	−1.97	0.55	0.28
	Treatment	0.66	−1.59	2.92	0.57
	Interaction of time × treatment	−0.49	−3.01	2.03	0.70
AMMI (kg/m^2^)	Moment	−0.45	−1.51	0.60	0.41
	Treatment	0.15	−2.69	2.99	0.92
	Interaction of time × treatment	0.46	−1.65	2.57	0.67
BBM thickness—affected side (mm)	Moment	−2.29	−4.34	−0.25	0.03
	Treatment	2.00	−3.28	7.28	0.46
	Interaction of time × treatment	1.47	−2.62	5.56	0.48
BBM thickness—unaffected side (mm)	Moment	−0.17	−1.05	0.71	0.70
	Treatment	2.90	−2.32	8.12	0.28
	Interaction of time × treatment	0.92	−0.83	2.68	0.30
RFM thickness—affected side (mm)	Moment	−1.45	−2.76	−0.14	0.03
	Treatment	−0.42	−4.80	3.95	0.85
	Interaction of time × treatment	−0.74	−3.36	1.88	0.58
RFM thickness—unaffected side (mm)	Moment	−0.71	−1.61	0.19	0.12
	Treatment	0.53	−3.81	4.87	0.81
	Interaction of time × treatment	0.06	−1.74	1.85	0.95
3MH—labeled isotope (%/h)	Moment	−0.00	−0.01	0.01	0.67
	Treatment	−0.00	−0.03	0.03	0.95
	Interaction of time × treatment	−0.01	−0.03	0.00	0.13
3MH—ELISA (nmol/mL)	Moment	−1.71	−11.9	8.47	0.74
	Treatment	−7.15	−36.2	21.88	0.63
	Interaction of time × treatment	−11.79	−32.0	8.44	0.25

FFMI: fat free mass index; AMMI: appendicular muscle mass index; BBM: biceps brachii muscle; RFM: rectus femoris muscle; 3MH: 3-Methylhistidine; and ELISA: enzyme-linked immunoassay. Performed generalized mixed models using normal and gamma distributions.

## Data Availability

The corresponding author may provide anonymized data for research purposes. The author may also email any additional information or data required to support the protocol. The data are not publicly available due to privacy.

## References

[1] GBD 2015 Mortality and Causes of Death Collaborators (2016). Global, regional, and national life expectancy, all-cause mortality, and cause-specific mortality for 249 causes of death, 1980–2015: A systematic analysis for the Global Burden of Disease Study 2015. Lancet.

[2] Katan M., Luft A. (2018). Global Burden of Stroke. Semin. Neurol..

[3] Ali S., Garcia J.M. (2014). Sarcopenia, cachexia and aging: Diagnosis, mechanisms and therapeutic options—A mini-review. Gerontology.

[4] Carin-Levy G., Greig C., Young A., Lewis S., Hannan J., Mead G. (2006). Longitudinal changes in muscle strength and mass after acute stroke. Cerebrovasc. Dis..

[5] Jørgensen L., Jacobsen B.K. (2001). Changes in muscle mass, fat mass, and bone mineral content in the legs after stroke: A 1 year prospective study. Bone.

[6] Ryan A.S., Buscemi A., Forrester L., Hafer-Macko C.E., Ivey F.M. (2011). Atrophy and intramuscular fat in specific muscles of the thigh: Associated weakness and hyperinsulinemia in stroke survivors. Neurorehabilit. Neural Repair..

[7] Ryan A.S., Dobrovolny C.L., Smith G.V., Silver K.H., Macko R.F. (2002). Hemiparetic muscle atrophy and increased intramuscular fat in stroke patients. Arch. Phys. Med. Rehabil..

[8] Scherbakov N., von Haehling S., Anker S.D., Dirnagl U., Doehner W. (2013). Stroke induced Sarcopenia: Muscle wasting and disability after stroke. Int. J. Cardiol..

[9] Scherbakov N., Sandek A., Doehner W. (2015). Stroke-related sarcopenia: Specific characteristics. J. Am. Med. Dir. Assoc..

[10] Scherbakov N., Pietrock C., Sandek A., Ebner N., Valentova M., Springer J., Schefold J.C., von Haehling S., Anker S.D., Norman K. (2019). Body weight changes and incidence of cachexia after stroke. J. Cachexia Sarcopenia Muscle.

[11] Perasso L., Spallarossa P., Gandolfo C., Ruggeri P., Balestrino M. (2013). Therapeutic use of creatine in brain or heart ischemia: Available data and future perspectives. Med. Res. Rev..

[12] Gualano B., Rawson E.S., Candow D.G., Chilibeck P.D. (2016). Creatine supplementation in the aging population: Effects on skeletal muscle, bone and brain. Amino Acids.

[13] Dolan E., Artioli G.G., Pereira R.M.R., Gualano B. (2019). Muscular Atrophy and Sarcopenia in the Elderly: Is There a Role for Creatine Supplementation?. Biomolecules.

[14] Candow D.G., Forbes S.C., Kirk B., Duque G. (2021). Current Evidence and Possible Future Applications of Creatine Supplementation for Older Adults. Nutrients.

[15] Izquierdo M., Ibañez J., González-Badillo J.J., Gorostiaga E.M. (2002). Effects of creatine supplementation on muscle power, endurance, and sprint performance. Med. Sci. Sports Exerc..

[16] Cordingley D.M., Cornish S.M., Candow D.G. (2022). Anti-Inflammatory and Anti-Catabolic Effects of Creatine Supplementation: A Brief Review. Nutrients.

[17] Kurzepa J., Kurzepa J., Golab P., Czerska S., Bielewicz J. (2014). The significance of matrix metalloproteinase (MMP)-2 and MMP-9 in the ischemic stroke. Int. J. Neurosci..

[18] Chen F., Lam R., Shaywitz D., Hendrickson R.C., Opiteck G.J., Wishengrad D., Liaw A., Song Q., Stewart A.J., Cummings C.E. (2011). Evaluation of early biomarkers of muscle anabolic response to testosterone. J. Cachexia Sarcopenia Muscle.

[19] Cardoso A.L., Fernandes A., Aguilar-Pimentel J.A., de Angelis M.H., Guedes J.R., Brito M.A., Ortolano S., Pani G., Athanasopoulou S., Gonos E.S. (2018). Towards frailty biomarkers: Candidates from genes and pathways regulated in aging and age-related diseases. Ageing Res. Rev..

[20] Crossland H., Smith K., Atherton P.J., Wilkinson D.J. (2020). A novel stable isotope tracer method to simultaneously quantify skeletal muscle protein synthesis and breakdown. Metabol. Open.

[21] de Souza J.T., Minicucci M.F., Ferreira N.C., Polegato B.F., Okoshi M.P., Modolo G.P., Phillips B.E., Atherton P.J., Smith K., Wilkinson D. (2023). Influence of CReatine supplementation on mUScle mass and strength after stroke (ICaRUS Stroke Trial): Study protocol for a randomized controlled trial. Trials.

[22] Bernhardt J., Churilov L., Ellery F., Collier J., Chamberlain J., Langhorne P., Lindley R.I., Moodie M., Dewey H., Thrift A.G. (2016). AVERT Collaboration Group. Prespecified dose-response analysis for A Very Early Rehabilitation Trial (AVERT). Neurology.

[23] Winstein C.J., Stein J., Arena R., Bates B., Cherney L.R., Cramer S.C., Deruyter F., Eng J.J., Fisher B., Harvey R.L. (2016). Guidelines for Adult Stroke Rehabilitation and Recovery: A Guideline for Healthcare Professionals From the American Heart Association/American Stroke Association. Stroke.

[24] Brasil (2014). Ministério da Saúde. Secretaria de Atenção à Saúde. Departamento de Atenção Básica. Guia alimentar para a população brasileira/Ministério da Saúde, Secretaria de Atenção à Saúde, Departamento de Atenção Básica, 2nd ed., 1 reimpr, Brasília: Ministério da Saúde. https://www.gov.br/saude/pt-br/assuntos/saude-brasil/publicacoes-para-promocao-a-saude/guia_alimentar_populacao_brasileira_2ed.pdf/view.

[25] Cincura C., Pontes-Neto O.M., Neville I.S., Mendes H.F., Menezes D.F., Mariano D.C., Pereira I.F., Teixeira L.A., Jesus P.A., de Queiroz D.C. (2009). Validation of the National Institutes of Health Stroke Scale, modified Rankin Scale and Barthel Index in Brazil: The role of cultural adaptation and structured interviewing. Cerebrovasc. Dis..

[26] Cruz-Jentoft A.J., Bahat G., Bauer J., Boirie Y., Bruyère O., Cederholm T., Cooper C., Landi F., Rolland Y., Sayer A.A. (2019). Writing Group for the European Working Group on Sarcopenia in Older People 2 (EWGSOP2), and the Extended Group for EWGSOP2. Sarcopenia: Revised European consensus on definition and diagnosis. Age Ageing.

[27] Smith-Ryan A.E., Fultz S.N., Melvin M.N., Wingfield H.L., Woessner M.N. (2014). Reproducibility and validity of A-mode ultrasound for body composition measurement and classification in overweight and obese men and women. PLoS ONE.

[28] Eickemberg M., Oliveira C.C., RORIZ A.K.C., Sampaio L.R. (2011). Bioelectric impedance analysis and its use for nutritional assessments. Rev. Nutr..

[29] Barry E., Galvin R., Keogh C., Horgan F., Fahey T. (2014). Is the Timed Up and Go test a useful predictor of risk of falls in community dwelling older adults: A systematic review and meta-analysis. BMC Geriatr..

[30] Jones C.J., Rikli R.E., Beam W.C. (1999). A 30-s chair-stand test as a measure of lower body strength in community-residing older adults. Res. Q. Exerc. Sport.

[31] Pinto E.B., Maso I., Vilela R.N., Santos L.C., Oliveira-Filho J. (2011). Validation of the EuroQol quality of life questionnaire on stroke victims. Arq. Neuropsiquiatr..

[32] Gotshalk L.A., Volek J.S., Staron R.S., Denegar C.R., Hagerman F.C., Kraemer W.J. (2002). Creatine supplementation improves muscular performance in older men. Med. Sci. Sports Exerc..

[33] Lanhers C., Pereira B., Naughton G., Trousselard M., Lesage F.X., Dutheil F. (2015). Creatine Supplementation and Lower Limb Strength Performance: A Systematic Review and Meta-Analyses. Sports Med..

[34] Balch M.H.H., Nimjee S.M., Rink C., Hannawi Y. (2020). Beyond the Brain: The Systemic Pathophysiological Response to Acute Ischemic Stroke. J. Stroke.

[35] Finnerty C.C., Mabvuure N.T., Ali A., Kozar R.A., Herndon D.N. (2013). The surgically induced stress response. JPEN J. Parenter. Enteral. Nutr..

[36] Chitramuthu B.P., Bennett H.P.J., Bateman A. (2017). Progranulin: A new avenue towards the understanding and treatment of neurodegenerative disease. Brain.

[37] Gualano B., Macedo A.R., Alves C.R., Roschel H., Benatti F.B., Takayama L., de Sá Pinto A.L., Lima F.R., Pereira R.M. (2014). Creatine supplementation and resistance training in vulnerable older women: A randomized double-blind placebo-controlled clinical trial. Exp. Gerontol..

[38] Forbes S.C., Candow D.G., Ostojic S.M., Roberts M.D., Chilibeck P.D. (2021). Meta-Analysis Examining the Importance of Creatine Ingestion Strategies on Lean Tissue Mass and Strength in Older Adults. Nutrients.

[39] Wang C.C., Fang C.C., Lee Y.H., Yang M.T., Chan K.H. (2018). Effects of 4-Week Creatine Supplementation Combined with Complex Training on Muscle Damage and Sport Performance. Nutrients.

[40] Lambertsen K.L., Finsen B., Clausen B.H. (2019). Post-stroke inflammation-target or tool for therapy?. Acta Neuropathol..

[41] Kato E.T., Morrow D.A., Guo J., Berg D.D., Blazing M.A., Bohula E.A., Bonaca M.P., Cannon C.P., de Lemos J.A., Giugliano R.P. (2023). Growth differentiation factor 15 and cardiovascular risk: Individual patient meta-analysis. Eur. Heart J..

[42] Swift I.J., Rademakers R., Finch N., Baker M., Ghidoni R., Benussi L., Binetti G., Rossi G., Synofzik M., Wilke C. (2024). A systematic review of progranulin concentrations in biofluids in over 7000 people-assessing the pathogenicity of GRN mutations and other influencing factors. Alzheimer’s Res. Ther..

[43] Liu C.J., Bosch X. (2012). Progranulin: A growth factor, a novel TNFR ligand and a drug target. Pharmacol. Ther..

[44] Horinokita I., Hayashi H., Oteki R., Mizumura R., Yamaguchi T., Usui A., Yuan B., Takagi N. (2019). Involvement of Progranulin and Granulin Expression in Inflammatory Responses after Cerebral Ischemia. Int. J. Mol. Sci..

[45] Lasek-Bal A., Jedrzejowska-Szypulka H., Student S., Warsz-Wianecka A., Zareba K., Puz P., Bal W., Pawletko K., Lewin-Kowalik J. (2019). The importance of selected markers of inflammation and blood-brain barrier damage for short-term ischemic stroke prognosis. J. Physiol. Pharmacol..

[46] Xie S., Lu L., Liu L., Bi G., Zheng L. (2016). Progranulin and short-term outcome in patients with acute ischaemic stroke. Eur. J. Neurol..

[47] Rasmussen R.S., Østergaard A., Kjær P., Skerris A., Skou C., Christoffersen J., Seest L.S., Poulsen M.B., Rønholt F., Overgaard K. (2016). Stroke rehabilitation at home before and after discharge reduced disability and improved quality of life: A randomised controlled trial. Clin. Rehabil..

